# Inflammatory Ratios as Predictors for Tumor Invasiveness, Metastasis, Resectability and Early Postoperative Evolution in Gastric Cancer

**DOI:** 10.3390/curroncol29120724

**Published:** 2022-11-27

**Authors:** Vlad I. Nechita, Nadim Al-Hajjar, Emil Moiş, Luminiţa Furcea, Mihaela A. Nechita, Florin Graur

**Affiliations:** 1 Department of Medical Informatics and Biostatistics, “Iuliu Hațieganu” University of Medicine and Pharmacy, Louis Pasteur Str., No. 6, 400349 Cluj-Napoca, Romania; 2 “Octavian Fodor” Regional Institute of Gastroenterology and Hepatology, Croitorilor Str., No. 19–21, 400162 Cluj-Napoca, Romania; 3 Department of Surgery, “Iuliu Hațieganu” University of Medicine and Pharmacy, Croitorilor Str., No. 19–21, 400162 Cluj-Napoca, Romania; 4 “Ion Chiricuță” Oncology Institute, Republicii Str., No.34–36, 400015 Cluj-Napoca, Romania

**Keywords:** gastric cancer, curative surgery, palliative treatment, inflammatory biomarkers, neutrophil-to-lymphocyte ratio (NLR), platelet-to-lymphocyte ratio (PLR), lymphocyte-to-monocyte ratio (LMR), systemic immune-inflammation index (SII)

## Abstract

Our study aimed to evaluate the baseline neutrophil-to-lymphocyte ratio (NLR), platelet-to-lymphocyte ratio (PLR), lymphocyte-to-monocyte ratio (LMR), and systemic immune-inflammation index (SII) in relation to invasion, metastasis, and resectability for patients with gastric cancer, respectively, as predictors of death during hospitalization or surgical complications. A retrospective cohort study was conducted on 657 gastric cancer subjects. Inflammatory biomarkers were computed. The associations with tumor stage, metastasis, optimal procedure, in-hospital mortality, and surgical complications were evaluated. Subjects who underwent curative-intent surgery presented lower median NLRs (2.9 vs. 3.79), PLRs (166.15 vs. 196.76), and SIIs (783.61 vs. 1122.25), and higher LMRs (3.34 vs. 2.9) than those who underwent palliative surgery. Significantly higher NLRs (3.3 vs. 2.64), PLRs (179.68 vs. 141.83), and SIIs (920.01 vs. 612.93) were observed for those with T3- and T4-stage cancer, in comparison with those with T1- and T2-stage cancer. Values were significantly higher in the case of metastasis for the NLR (3.96 vs. 2.93), PLR (205.22 vs. 167.17), and SII (1179 vs. 788.37) and significantly lower for the LMR (2.74 vs. 3.35). After the intervention, the NLR, PLR, and SII values were higher (*p* < 0.01) for patients with surgical complications, and the NLR and SII values were higher for those who died during hospitalization. Higher NLRs, PLRs, SIIs, and lower LMRs were associated with a more aggressive tumor; during early follow-up, these were related to post-operative complications and death during hospitalization.

## 1. Introduction

Gastric cancer is the 5th most frequent type of neoplasia (after lung, breast, colorectal, and prostate cancer) and the 3rd cause of death due to neoplasia (after lung and colorectal cancers) [1,2]. This type of cancer is about two times more frequent in the male population, with a difference between developed countries (2.2 times more frequent in males) and developing countries (1.83 times more frequent in males), but always has a higher prevalence in men than in women [1,2,3]. The incidence of gastric neoplasia has decreased worldwide as a result of the treatment of *Helicobacter pylori* infection, better eating habits, and food preservation methods [1,3]. The five-year survival rate is about 20% in most countries [3,4].

The relationship between inflammation and neoplasia was suggested by Rudolf Virchow, who observed the infiltration of leucocytes into tumors [5,6]. Several inexpensive inflammatory biomarkers called composite ratios, such as the neutrophil-to-lymphocyte ratio (NLR), platelets-to-lymphocyte ratio (PLR), lymphocyte-to-monocyte ratio (LMR), and systemic immune-inflammation index (SII), are easily analyzed in the perioperative period in cancer patients [7]. A high NLR was mainly reported to be associated with a decrease in survival [8,9,10,11,12]. For patients with resectable gastric cancer, significantly higher NLR values were reported in men (median = 3.00, IQR = (0.24 to 30.3); *p* < 0.01) [8], in older patients (≥66 years, median = 3.00, IQR = (2.11 to 4.10); *p* < 0.01) [8], in those with vascular, lymphatic but not perineural microscopic invasion [8,10], when the patient’s tumor size was >4.8 cm [10], for higher T stages, metastasis and positive resection margins [10]. A significantly higher NLR value was also observed for unresectable patients with peritoneal metastasis (*p* = 0.041) and this is also related to the number of metastasis sites [12].

Lian et al. evaluated both the NLR and PLR in a study of resectable gastric cancer patients that were divided into two groups according to their preoperative NLR (<4.02, ≥4.02) and PLR (<208, ≥208) values. Overall survival was significantly better for patients with lower NLR (*p* < 0.004) and PLR (*p* < 0.008) values. Highly significant (*p* < 0.001) was also the association of high NLR, respectively high PLR values with higher T stage (T3, T4) and higher N stage (N2, N3). The average PLR (140.25 ± 24.62) and NLR (2.18 ± 0.31) values in healthy subjects were significantly lower (*p* < 0.001) than PLR (207.82 ± 50.71) and NLR (3.97 ± 0.53) values for gastric cancer subjects [13]. A meta-analysis showed that the group with a PLR higher than 150 presented a higher rate of lymph node metastasis, serosa invasion, and higher stage of cancer (stage III and IV). PLR was not associated with tumor size, nor grading or localization (cardia vs. non-cardia) [14].

It was reported that NLRs and PLRs have better diagnostic significance (*p* < 0.0001) for early-stage gastric cancer patients than the classical markers, such as CEA (carcinoembryonic antigen, cut-off value of 2.1, AUC (area under the curve) of 0.623; Sp of 70.59) and CA19-9 (carbohydrate antigen, cut-off value of 25.1, AUC of 0.565; Sp of 93.13), with a cut-off value of 2.25 for the NLR (AUC of 0.715; Sp of 83.04%) and 147.368 for PLR (AUC of 0.707; Sp of 81.79%). The sensitivity was poor in both cases (48.88% for NLR and 48.20% for PLR). When combined, the AUC for NLR and PLR was 0.739, respectively, and the Sp was 85.65%. The cut-off values for both ratios were lower in males than in females [15].

A low lymphocyte-to-monocyte ratio (LMR) was also an independent risk factor for shorter survival (HR = 1.49; 95%CI 1.17–1.89; *p* = 0.001) in stage I–III gastric cancer patients, along with older age, a higher TNM stage and lack of chemotherapy treatment [16]. A meta-analysis of 4908 patients also found that a low LMR was associated with decreased overall survival (*p* < 0.001), older age (*p* < 0.001), male gender (*p* < 0.001), a CEA > 5 ng/mL (*p* < 0.001), tumor size > 3 cm (*p* = 0.04), III and IV TNM stage (*p* = 0.02), positive lymph nodes (*p* < 0.001) and metastasis (*p* = 0.007) [17].

The SII computed using the formula SII = Platelets*Neutrophils/Lymphocytes was also used as a prognostic factor in gastric cancer patients with a cut-off value of 390 × 10^9^ cells/L. Patients with a low SII had better tumor differentiation (*p* = 0.002) and better one-year survival rates (*p* = 0.006), but no association with age or Ki-67 expression was found [18].

All of the presented inflammatory biomarkers seem to be related to some of the tumor characteristics; furthermore, they should be evaluated in the context of early and late complications that might have a significant influence on prognosis. In Romania, a prospective study was conducted on 204 patients to evaluate the preoperative NLR as a prognosis factor for anastomotic leakage after surgery for gastric cancer. NLR was calculated as a ratio of the percentages from peripheral blood cells with a range from 1.49 to 9.75 and an average of 2.71 ± 1.16. The group (22 patients) with higher NLR values (≥3.54) had a higher rate of anastomotic fistula (*p* < 0.001) and mortality (*p* = 0.025) [19]. Another similar study was conducted by Molnar et al. over a period of 6 years (178 patients), considering the NLR, PLR, and LMR (upon presentation) with regard to anastomotic complications (fistula and stenosis). An increased platelet number (*p* = 0.043, Mann–Whitney test) and higher PLR (*p* = 0.023, Mann–Whitney test) were observed for patients who developed stenosis. No differences were observed in the NLR or PLR for patients with fistulas [20].

The aim of this study was to evaluate inflammatory biomarkers, such as NLR, PLR, LMR and SII, and the outcome of gastric cancer patients depending on tumor-related factors, such as tumor stage (T stage), tumor invasion of one or more organs, presence and number of metastasis sites (M), and also depending on suitable treatment, including curative-intent surgery or palliative surgical procedures. Inflammatory ratios were also evaluated at early follow-up after surgery (4th to 5th day) for the association with early post-operative surgical complications and death during the hospitalization period.

## 2. Materials and Methods

### 2.1. Setting and Study Design

We conducted a retrospective cohort study on patients with gastric cancer from the surgery department at the “Prof. Dr. Octavian Fodor” Regional Institute of Gastroenterology and Hepatology, Cluj-Napoca, Romania, between 1 January 2016 and 31 December 2019.

This study received approval (approval No. 121/24.04.2019) from the “Iuliu Hațieganu” Ethics Committee and approval (approval No. 8900/10.07.2019) from the Ethics Committee of the “Prof. Dr. Octavian Fodor” Regional Institute of Gastroenterology and Hepatology.

### 2.2. Participants

All patients with a diagnostic of gastric cancer (Ro-DRG, diagnosis-related groups; codes: C16.0–C16.9) following their visit to the surgical department and histopathological confirmation of gastric malignancy were eligible for enrollment.

**Figure 1:**
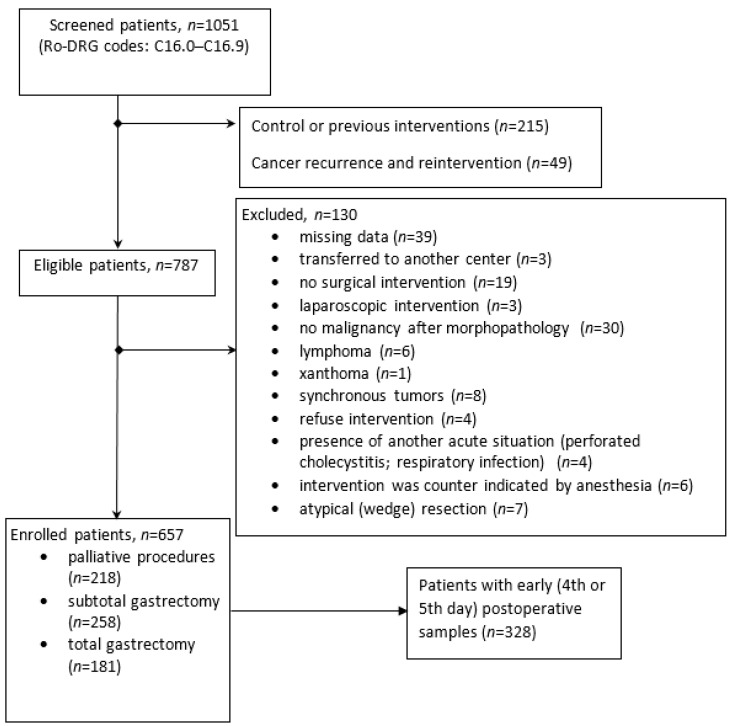
Flow-chart with subjects enrolled in the study.

The cohort was divided into groups according to the surgical procedure, including curative-intent gastrectomy (total or partial gastrectomy) and palliative intervention (Figure 1). Patients with a poor condition and advanced tumors (local invasion or metastasis) received only palliative surgical treatment, including cytoreductive surgery, metastasectomy, biopsy, devascularization, feeding stoma, or digestive anastomosis. Eight subjects with tumor and metastasis resection were also considered in the palliative group. Surgical complications were only considered as documented complications related to the procedure, and not medical complications (bronchopneumonia, clostridium infection, arrhythmia, pancreatitis, or urinary tract infections).

Only patients without previous surgical treatment were enrolled in the study; those who had undergone atypical resection curative surgery, as well as those with records of relapse and reintervention, were excluded. Gastric cancer diagnosis and staging were established based on biopsy, a pathology report, intraoperative findings, and surgical protocols, including medical imaging (ultrasonography, computer tomography, magnetic resonance, and echo-endoscopy).

### 2.3. Data Source and Collection

The hospital database and patients’ observation sheets were consulted and used for data collection. Demographic information (e.g., age, sex and setting), surgery information, and operative protocols (e.g., type of intervention, anastomosis, tumor extension, and multi-organ involvement, macroscopic invasion, occurrence and localization of metastasis, complications and death during hospitalization period), pathology information (e.g., tumor type and staging and invasion), laboratory findings (e.g., absolute number of neutrophils (10^3^/μL), lymphocytes (10^3^/μL), monocytes (10^3^/μL) and platelets (10^3^/μL) were collected.

For all the patients, the numbers of neutrophils, lymphocytes, monocytes, and platelets were collected at presentation (baseline), and, when available, the values were collected at 96–120 h after intervention (on the 4th, or 5th day after surgery). The NLR, PLR, and LMR values before and after surgical intervention were calculated by dividing the absolute values of peripheral cells (from venous blood). The SII was computed using the product between the number of platelets and neutrophils divided by the lymphocyte number.

### 2.4. Statistical Methods

For statistical analysis, the Statistica program (v. 13, StatSoft, Tulsa, OK, USA) was used. Shapiro–Wilk tests, Kolmogorov–Smirnov tests, skewness, and kurtosis were used to assess quantitative data distribution. For the quantitative variables, the mean and standard deviation (in case of normal distribution) or median and ranges (for non-normal distribution) were calculated; Mann–Whitney and Kruskal–Wallis tests for the independent groups were used when we compared two and more than two groups, respectively. A significance level of 5% was considered and a *p*-value less than 0.05 was considered as statistically significant. Our hypothesis was that preoperative NLRs, PLRs, LMRs, and SIIs were significantly different depending on surgery type (curative-intent or palliative surgery), tumor stage and invasion of other organs (T), presence, and the occurrence of metastasis (M). Post-operative inflammatory ratios were also evaluated for surgical complications and death during the hospitalization period.

## 3. Results

One thousand and fifty-one medical charts from patients with gastric cancer were reviewed. Eight hundred and thirty-six patients were eligible for the study, and six hundred and fifty-seven were included in the analysis. The details of the patients eligible for this study’s inclusion and exclusion criteria can be observed in the flowchart (Figure 1).

Resection was indicated for 439 patients (66.81%) and palliative procedures for 218 (33.18%). The age of the participants was between 30 and 94 years old, with a male-to-female ratio of 2.01 and an average of 65.21 ± 11.00 years. A percentage of 57.38% of the patients lived in rural settings. The characteristics of the included subjects can be found in Table 1.

**Figure 2:**
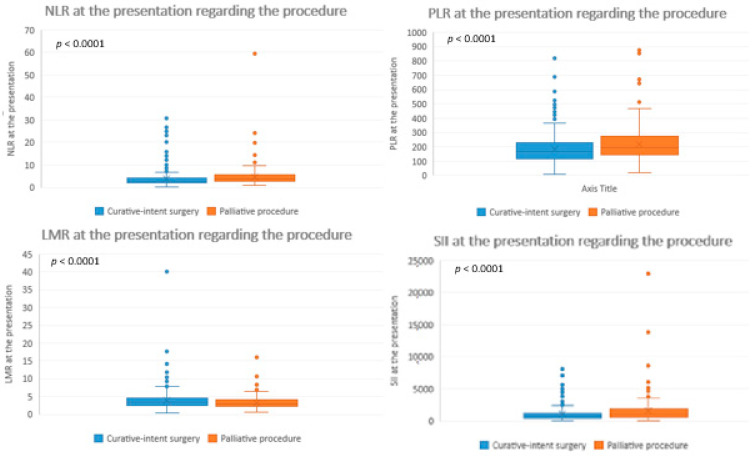
Inflammatory biomarkers at presentation regarding the procedure. NLR = neutrophil-to-lymphocyte ratio; PLR = platelet-to-lymphocyte ratio; LMR = lymphocyte-to-monocyte ratio; SII = systemic immune-inflammation index.

A statistically significant higher NLR, PLR and SII, and lower LMR were observed at presentation for the patient candidates who underwent palliative surgical procedures in comparison with those who underwent curative-intent resection (Table 2 and Figure 2).

A statistically significant difference was observed for the inflammatory ratios regarding the T-stage classes (Table 3). When comparing low-T stage subjects (T1 and T2) with high-T stage subjects (T3 and T4), significantly higher NLRs, PLRs and SIIs, and lower LMRs were observed for advanced cases. No statistically significant differences (*p* > 0.1, Mann–Whitney U test) were observed between the T1 and T2 stages for any of the inflammatory ratios, nor between the T2 and T3 stages. A significantly higher NLR (*p* < 0.0001, Mann–Whitney test), PLR (*p* < 0.0001, Mann–Whitney test), and SII (*p* < 0.0001, Mann–Whitney test) and a significantly lower LMR (*p* = 0.0129, Mann–Whitney test) were observed for patients with T4-stage cancer in comparison with T3 stage.

Statistically significant higher NLR, PLR and SII values, and lower LMR values were observed for patients with metastasis, and no differences regarding the number of metastatic organs were found (Table 4).

Statistically significant higher NLR, PLR and SII values and lower LMR values were observed for patients with macroscopic invasion, and no differences regarding the number of organs involved were reported (Table 5).

Thirty-eight subjects (5.78%) with mentioned surgical complications were identified retrospectively, which were as follows: twenty-one cases of fistulae (55.26%), eight cases of surgical wound suppurations (21.05%), five evacuated hematomas or hemoperitoneum (13.15%), three peritoneal abscesses (7.89%) and one case of evisceration (2.63%). No association between the pre-operative inflammatory ratios and surgical complications or death during the hospitalization period was observed (Table 6), but the association was significant between the early follow-up values and death, respectively surgical complications.

Higher NLR and SII values and lower LMR values on the 4th or 5th post-operative day were observed for subjects with surgical complications and subjects who died during the hospitalization period. A higher PLR was associated with surgical complications, but not with death during the hospitalization period (Table 7).

**Figure 3:**
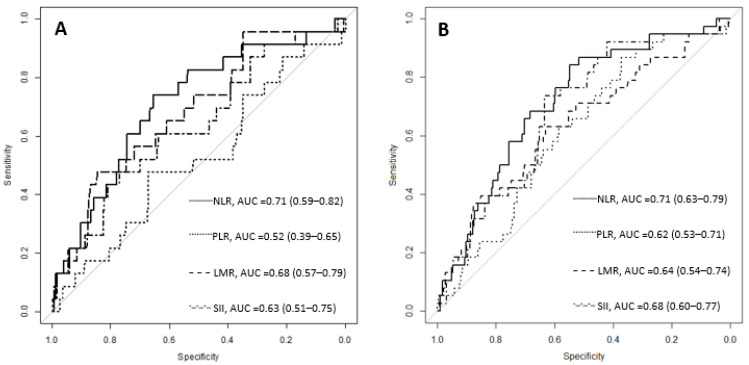
The ROC curves for inflammatory ratios considering death during the hospitalization period (**A**) and surgical complications (**B**). NLR = neutrophil-to-lymphocyte ratio; PLR = platelet-to-lymphocyte ratio; LMR = lymphocyte-to-monocyte ratio; SII = systemic immune-inflammation index; AUC = area under the curve, with 95% confidence intervals.

The ROC (receiver operating characteristic) curve analysis revealed the post-operative NLR, PLR, LMR and SII cut-off values for death during the hospitalization period (7.07; 279.19; 1.1 and 2489.79) and the cut-off values for surgical complications (6.93; 183.7; 1.73 and 1592.18). The AUC (area under the curve) with 95% confidence intervals is presented in Figure 3.

## 4. Discussion

This study improves gastric cancer prognostic models by including biological prognostic factors in addition to factors related to the anatomical extension of the tumor. In our study, we evaluated 657 patients with I–IV stage gastric cancer from a single tertiary center in Romania to investigate prognostic indicators that are very accessible by routine blood examinations. Prognostic indicators for unresectable tumors following palliative surgery are not widely studied in the literature.

The TNM stage (tumor-node metastasis) indicates the tumor behavior and can be considered the most important prognostic factor for gastric cancer [21], but because of the different prognoses between similar-stage subjects, this method is not a precise outcome predictor [10]. Apart from anatomic extent classification, cancer biology and inflammatory characteristics can offer important contributions [22]. Some supplementary biomarkers can be considered to improve personalized, targeted therapies. Inflammatory biomarkers managed to show their utility by not only increasing awareness in the early stage of gastric cancer [15], but also as prognostic factors.

The subjects with recurrent disease were excluded from our analysis (Figure 1); in the literature, differences have been reported, and the NLR seems to be higher for advanced unresectable gastric cancer patients at first presentation compared to those who reported relapse [12]. Subjects treated with neoadjuvant therapy were not excluded from the analysis. According to Wang et al., neoadjuvant treatment produces a decrease in both the neutrophil and lymphocyte lines, but without significant changes in NLRs (*p* = 0.86) [8].

Tumor aggressiveness and metastasis behavior are influenced by the microenvironment, which plays a role in the neoformation process [23,24]. The concept of polarization and immune cells’ dual role is mentioned in the literature; they can drive specific metabolic pathways due to their anti-tumorigenic or pro-tumorigenic properties [25]. Neutrophils can increase the level of nitric oxide (known to increase the rate of cellular mutations), arginase, and reactive oxygen species in the extracellular matrix and reduce the T lymphocyte immune response and also can increase IL8 (interleukin-8) levels and promote angiogenesis [26]. They are also considered as carriers of VEGF (vascular endothelial growth factor), so neutrophils can promote tumor growth and metastasis and are associated with more advanced and aggressive tumors [27,28,29]. Lymphocytes, on the other hand, exhibit anti-tumor activity and a low number is related to a weak immune response [30]. Platelets can promote tumor cells’ survival in circulation and increase tumor emboli [31]. Some experimental models have been used to prevent metastasis by lowering the number of platelets [31,32].

The risk of gastric cancer is significantly higher for men and has increased over time from 1.86 (1990) to 2.20 (2017), with unimportant differences until the age of 44 and a peak of 2.74 between 65 and 69 years old [1,2,3,33], which is consistent with the ratio of 2.01 for our study cohort. A higher male frequency was observed for all the subgroups (Table 1). The rate for R1 resection described in the literature [34] varies between 1.8 and 9% and a slightly higher R1 rate of 13.21% was observed in our study cohort.

Information regarding the surgical procedures was collected for total gastrectomy, partial gastrectomy, and palliative surgery. Between total and partial gastrectomy, no differences were found regarding the preoperative inflammatory ratios (only regarding the platelets’ absolute number), so we considered them together for analysis as a gastrectomy group. Candidates for palliative procedures presented significantly higher NLR, PLR, and SII values and lower LMR values than subjects who underwent surgical resection of their tumor (Table 2, Figure 2). Decision regarding the optimal procedure is influenced by local invasion and metastasis. The inflammatory ratios were higher for the patients with a higher T stage, except for the LMR, which had a lower value. An association between NLR and T stage was described by Sahin et al. and it was reported that a higher NLR was associated with a higher T stage [35]. The results were comparable with our findings, as we reported an NLR median value of 2.8 for T1 vs. 2.79 in our cohort; 4.4 for T4 vs. 3.5 in our cohort. In their study, an ascending trendline was observed for the NLR value with the T stage and a significant difference was reported when comparing a lower T stage with a higher T stage. In our cohort, patients with T2- and T3-stage cancer presented lower values than those with T1, but overall, when comparing a low T stage (1 + 2) vs. a high T stage (3 + 4), the NLR value was statistically significantly higher (*p* = 0.00038, Man–Whitney test). There was also a significant difference in the NLR between individual T groups (*p* < 0.001, Kruskal–Wallis test). Another study from the same center in Romania on Klatskin tumors did not find any significant differences for the inflammatory ratios at baseline regarding the presence of invasion [36]. Hsu et al. divided 1030 subjects with resectable gastric cancer into 2 groups with low NLR values of ≤ 3.44 and high NLR values of > 3.44. The high NLR group presented a higher proportion of large tumors (>4.8 cm) and T4-stage cancer [10]. The PLR followed the NLR pattern, increasing with tumor stage (Table 3). An analysis of 49 studies (28929 patients) showed that higher PLR values (cut-off = 150) were associated with a higher rate of lymph node metastasis (OR = 1.17, 95%CI 1.02–1.33, *p* = 0.023), a higher rate of serosa invasion (T3 and T4) (OR = 1.34, 95%CI 1.10–1.64, *p* = 0.003) and a higher cancer stage (stage III and IV) (OR = 1.20, 95%CI 1.06–1.37, *p* = 0.004). The PLR association with tumor size, grading, or localization (cardia vs. non-cardia) was inconclusive [14]. The LMR decreased for patients with advanced stages of cancer in our study. This can be explained by the elevation of neutrophils and polymorphonuclear inflammatory reactions that can reduce lymphocyte anti-tumor activity, inhibit activated T cells, and natural killer cells, and decrease lymphocyte cytolysis activity [37]. The advanced T4 stage can be divided into T4a, which refers to serosa invasion but no nearby organ involvement, and T4b, which refers to the involvement of nearby structures [38]. Even if inflammatory ratios have significantly higher values for subjects with invasion (T4b) (Table 5), the number of invasion sites makes no significant difference on the NLR, PLR, LMR, or SII. Apart from the general tendency of the inflammatory ratios to increase (decrease for LMR) with more advanced tumor characteristics, for the patients who suffered from the invasion of multiple organs, the ratios decreased in comparison with single-organ invasion (Table 5). This may be linked to a lowering in the immune response for terminal cases or a type of deficiency after an important immune response to tumor aggression for a long period. It is important to mention that both situations refer to the same T4b tumor stage with an increased inflammatory response, compared to the other T stages (Table 3).

A higher NLR was also associated with peritoneal metastasis (*p* = 0.041) for subjects with unresectable gastric cancer [12]. On the other hand, Sahin et al. found no difference in the NLR between metastatic and non-metastatic subjects (*p* = 0.55) [35], but the sample size was small (189 cases) with only 18 metastasis cases. A larger number of subjects with metastasis, 166 (25.26%) from a total of 657, was described in our study cohort. The inflammatory ratios had significantly higher values in the group with metastasis, except for the LMR, which had a significantly lower value. The NLR, PLR, LMR, and SII did not have significantly different values between patients with single-organ metastasis and multiple-organ metastasis (Table 4). The association of the NLR with the number of metastasis locations was described by Murakami et al. [12]. More frequent metastatic disease for NLR values greater than 3.44 was described by Hsu et al. [10]. The values for the inflammatory ratios at presentation were not associated with death during the hospitalization period nor with post-operative complications (Table 6), but a significant association was found between death during the hospitalization period or surgical complications, and the follow-up NLR, PLR, LMR and SII values (Table 7). Inflammatory ratios on the 4th or 5th post-operative day are more relevant to a patient’s evolution after the intervention. Mohri et al. reported a higher rate of complications (37%) for subjects with an NLR > 3 before curative-intent gastrectomy than subjects with an NLR ≤ 3 (23%). In the multivariate analysis, a higher age (>70 years old, *p* = 0.03), the proximal location of the tumor (0.02), and an NLR > 3 (*p* = 0.04) were independently related to post-operative infectious complications [39]. Dal et al. reported that a higher NLR was related to anastomosis leakage, early post-operative complications, and prolonged hospitalization for patients who underwent esophageal cancer resection [40]. Information related to early post-operative inflammatory ratios is scarce. Liu et al. reported significantly higher NLR values on post-operative day 3 (average of 10.9 and cut-off of 8.6) and 5 (average of 9.3 and cut-off of 5.5) for patients that suffered an anastomosis leak after rectal cancer resection. Differences in the absolute neutrophil count were not significant between the groups [41]. Similar results were reported for our study cohort, with a median NLR of 9 (9.66 to 13.45) for the group with perioperative mortality and 8.64 (6.29 to 12.89) for the group with surgical complications vs. 5.42 (3.63 to 7.91) for subjects without complications. When comparing the ROC curves between the inflammatory ratios, the best AUC was observed for the NLR (Figure 3), with a cut-off value of 6.93 for patients with surgical complications and 7.07 for those who died during the hospitalization period.

Full blood count tests are routinely performed for almost all patients on a surgical unit and do not require additional costs. In our study, the inflammatory ratios were evaluated at admission before a procedure was decided (resection or palliative surgery) and before medical treatment using the laboratory results available in our center. For some subjects, palliative treatment was decided intraoperatively due to metastasis or invasion.

The possible limits of our study are as follows: (1) comorbidities and chronic treatment influence were not considered; (2) the retrospective design; (3) the single center data collection; (4) the correspondence with other inflammatory markers, such as procalcitonin and C reactive protein, was not evaluated, as they are not routinely investigated; (5) for subjects who underwent palliative surgery, their pathology reports were not as detailed as for those with tumor excisions regarding lymph node involvement and other tumor characteristics; (6) lymph node stadialization (N) was not considered for evaluation; (7) the complications reported may have been underestimated due to retrospective data collection. Nevertheless, this study has some strengths, with the most important being the large sample on which the analysis was performed.

## 5. Conclusions

The NLR, PLR, LMR, and SII have the potential to be considered as prognosis biomarkers in gastric cancer patients before surgery and in the early follow-up period. A higher NLR, PLR, and SII and a lower LMR at admission were associated with characteristics of a more aggressive tumor extension. Higher values of the NLR, PLR, and SII and a lower LMR in the early post-operative period (day 4 or 5) were related to surgical complications and poor short-term prognosis. Patients with higher NLR, PLR and SII values and lower LMR values would benefit from more attention over the surveillance period and further adjuvant treatment. Larger cohorts and prospective studies are needed to confirm these results.

## Figures and Tables

**Table 1:** The main characteristics of the sample (*n* = 657).

	All Subjects	Total Gastrectomy	Partial Gastrectomy	Candidates for Curative Surgery	Palliative Procedures
	(*n* = 657)	(*n* = 181)	(*n* = 258)	(*n* = 439)	(*n* = 218)
Characteristics	Value (median, Q1, Q3)				
Age * (years) mean ± SD	65.21 ± 11.00	63.99 ± 11.40	65.52 ± 10.04	64.89 ± 10.64	65.84 ± 11.70
Gender, No. of males (%)	439 (66.81%)	126 (69.61%)	168 (65.11%)	294 (66.97%)	145 (66.51%)
Mechanical anastomosis		49 (27.07%)	22 (8.52%)		
Complications	No. (%)				
Death during hospitalization period	30 (4.56%)	13 (7.18%)	8 (3.1%)	21 (4.78%)	9 (4.12%)
Surgical complications	49 (7.45%)	13 (7.18%)	20 (7.75%)	33 (7.51%)	16 (7.33%)
Stadialization	No. (%)				
T1	53 (8.06%)	15 (8.28%)	38 (14.72%)	53 (12.07%)	0
T2	46 (7.00%)	13 (7.18%)	32 (12.4%)	45 (10.25%)	1 (0.45%)
T3	141 (21.46%)	52 (28.72%)	59 (22.86%)	111 (25.28%)	30 (13.76%)
T4	417 (63.47%)	101 (55.8%)	129 (50%)	230 (52.39%)	187 (85.77%)
M1	166 (25.26%)	0	0	0	166 (76.14%)
Metastasis in more than one organ	33 (5.02%)	0	0	0	33 (15.13%)
Macroscopic invasion of more than one organ	76 (11.56%)	16 (8.83%)	8 (3.10%)	24 (5.46%)	52 (23.85%)
Positive resection margin (R1)		34 (18.78%)	24 (9.30%)	58 (13.21%)	

Values are presented as absolute values and percentages from the total of each group; * the mean and standard deviation are presented for age.

**Table 2:** Inflammatory biomarkers at presentation regarding treatment groups.

	Gastrectomy (*n* = 439)	Palliative Procedures (*n* = 218)	*p*-Value	All Subjects (*n* = 657)
Neutrophils (10^3^/μL)	4.84 (3.73–6.2)	5.75 (4.52–7.38)	<0.0001	5.13 (3.93–6.61)
Lymphocytes (10^3^/μL)	1.67 (1.32–2.12)	1.55 (1.16–1.99)	0.008	1.65 (1.28–2.08)
Monocytes (10^3^/μL)	0.51 (0.39–0.66)	0.54 (0.41–0.7)	0.085	0.52 (0.4–0.67)
Platelets (10^3^/μL)	266 (215.5–344.5)	297 (230.25–383.5)	0.002	275 (219–354)
NLR baseline	2.9 (2.00–3.99)	3.79 (2.57–5.57)	<0.0001	3.14 (2.16–4.38)
PLR baseline	166.15 (118.12–227.63)	196.76 (142.24–271.2)	<0.0001	174.71 (122.98–247.72)
LMR baseline	3.34 (2.49–4.59)	2.9 (2.21–4.02)	<0.0001	3.19 (2.4–4.28)
SII baseline	783.61 (482.75–1270.83)	1122.25 (655.06–1845.91)	<0.0001	872.74 (531.62–1509.57)

Values are presented as median and interquartile range (Q1–Q3); NLR = neutrophil-to-lymphocyte ratio; PLR = platelet-to-lymphocyte ratio; LMR = lymphocyte-to-monocyte ratio; SII = systemic immune-inflammation index.

**Table 3:** Inflammatory biomarkers at presentation regarding T (tumor) stage (*n* = 657).

	T1 (*n* = 53)	T2 (*n* = 46)	T3 (*n* = 141)	T4 (*n* = 417)	*p*-Value
NLR	2.79 (1.88–3.96)	2.46 (1.81–3.36)	2.60 (1.60–3.57)	3.50 (2.556–4.83)	<0.0001
	2.64 (1.88–3.71)		3.30 (2.27–4.52)		0.0003
PLR	143.01 (95.02–180.34)	140.36 (106.5–206.26)	155.48 (108.59–207.63)	193.08 (132.77–268.81)	<0.0001
	141.83 (102.04–193.03)		179.68 (128.02–254.21)		<0.0001
LMR	3.55 (2.79–4.69)	3.87 (3.05–4.781)	3.44 (2.67–4.53)	3.02 (2.12–4.09)	0.004
	3.72 (2.79–4.73)		3.11 (2.32–4.22)		0.0007
SII	600.51 (416.35–945.00)	639.84 (355.02–947.46)	697.62 (333.48–941.44)	1080.56 (567.19–1762.53)	<0.0001
	612.93 (395.91–949.33)		920.01 (573.75–1603.45)		<0.0001

Values are presented as median and interquartile range (Q1–Q3); NLR = neutrophil-to-lymphocyte ratio; PLR = platelet-to-lymphocyte ratio; LMR = lymphocyte-to-monocyte ratio; SII = systemic immune-inflammation index. For each inflammatory ratio, the values are presented for individual T stages (Kruskal–Wallis test), and on the second row for T1–T2 vs. T3–T4 (Mann–Whitney test).

**Table 4:** The association between inflammatory ratios at presentation and presence of metastasis in one or more organs.

	M0 (*n* = 491)	M1 (*n* = 166)	*p*-Value	One Organ with Metastasis (*n* = 133)	Two or More Organs with Metastasis (*n* = 33)	*p*-Value
NLR	2.93 (2.04–4.01)	3.96 (2.64–5.59)	<0.0001	3.86 (2.6–5.64)	4.2 (2.73–4.83)	0.89
PLR	167.17 (118.93–233.28)	205.22 (148.63–279.78)	<0.0001	197.63 (150–270.54)	212.64 (139.37–288.37)	0.78
LMR	3.35 (2.51–4.47)	2.74 (2.16–4.02)	<0.0001	2.76 (2.12–4.02)	2.70 (2.19–4.06)	0.82
SII	788.37 (486.83–1289.13)	1179 (703.75–1845.91)	<0.0001	1163.32 (701.63–1809.54)	1354 (706.10–1898.52)	0.71

Values are presented as median and interquartile range (Q1–Q3); NLR = neutrophil-to-lymphocyte ratio; PLR = platelet-to-lymphocyte ratio; LMR = lymphocyte-to-monocyte ratio; SII = systemic immune-inflammation index.

**Table 5:** Inflammatory biomarkers at presentation regarding macroscopic invasion (*n* = 657).

	No Invasion (*n* = 500)	Macroscopic Invasion (*n* = 157)	*p*-Value	Invasion of One Organ (*n* = 81)	Invasion of More Organs (*n* = 76)	*p*-Value
NLR	2.94 (1.98–4.13)	3.78 (2.71–5.48)	<0.0001	4.16 (2.85–5.83)	3.63 (2.5–5.22)	0.07
PLR	167.15 (118.16–233.15)	212.92 (158.82–280)	<0.0001	235.34 (164.62–288.37)	202.02 (148.46–269.71)	0.06
LMR	3.30 (2.46–4.37)	2.94 (2.15–4.00)	0.0023	2.75 (2.03–3.82)	3.13 (2.21–4.02)	0.36
SII	813.55 (473.61–1302.77)	1171.48 (689.74–1828.58)	<0.0001	1360.21 (784.37–1898.52)	1095.01 (635.36–1705.55)	0.08

Values are presented as median and interquartile range (Q1–Q3); NLR = neutrophil-to-lymphocyte ratio; PLR = platelet-to-lymphocyte ratio; LMR = lymphocyte-to-monocyte ratio; SII = systemic immune-inflammation index.

**Table 6:** The association of inflammatory ratios at presentation with post-operative complications (death during the hospitalization period and surgical complications).

Baseline	Death during the Hospitalization Period (*n* = 30)	Alive (*n* = 627)	*p*-Value	Surgical Complications (*n* = 49)	Without Complications (*n* = 608)	*p*-Value
NLR presentation	3.94 (2.54–5.79)	3.13 (2.12–4.31)	0.05	3.46 (2.31–4.75)	3.12 (2.12–4.38)	0.37
PLR presentation	175.69 (115.97–267.47)	174.56 (123.16–247.22)	0.06	179.51 (122.98–252.89)	172.63 (122.96–247.41)	0.45
LMR presentation	2.91 (2.02–4.11)	3.21 (2.42–4.28)	0.07	3.12 (2.29–4.01)	3.20 (2.41–4.28)	0.35
SII presentation	952.29 (595.09–1800.92)	867.03 (527.49–1469.27)	0.2	949.27 (592.87–1762.53)	862.08 (527.80–1455.44)	0.17

Values are presented as median and interquartile range (Q1–Q3); NLR = neutrophil-to-lymphocyte ratio; PLR = platelet-to-lymphocyte ratio; LMR = lymphocyte-to-monocyte ratio; SII = systemic immune-inflammation index.

**Table 7:** The association of inflammatory ratios on the 4th and 5th follow-up day with post-operative complications (death during the hospitalization period and surgical complications) (*n* = 328) *.

	Death during the Hospitalization Period (*n* = 23)	Alive (*n* = 305 *)	*p*-Value	Surgical Complications (*n* = 38)	Without Complications (*n* = 290 *)	*p*-Value
NLR post-op	9.00 (6.66–13.45)	5.68 (3.69–8.57)	0.00078	8.64 (6.29–12.89)	5.42 (3.63–7.91)	0.000019
PLR post-op	223.08 (177.61–317.11)	218.42 (162.92–310.00)	0.7486	266.48 (203.89–329.29)	214.24 (159.08–309.79)	0.0209
LMR post-op	1.42 (1.02–2.14)	2.01 (1.41–2.83)	0.00355	1.57 (1.05–2.31)	2.03 (1.42–2.82)	0.00518
SII post-op	1976.74 (1124.76–3143.61)	1312.78 (823.81–2256.88)	0.0317	1776.19 (1458.01–3387.87)	1259.03 (768.19–2233.28)	0.000240

Values are presented as median and interquartile range (Q1–Q3); NLR = neutrophil-to-lymphocyte ratio; PLR = platelet-to-lymphocyte ratio; LMR = lymphocyte-to-monocyte ratio; SII = systemic immune-inflammation index. * Follow-up data were available only for 328 subjects.

## Data Availability

Not applicable.
